# Supplementary material for: Broadband, High-Reflectivity Dielectric Mirrors at Wafer Scale: Combining Photonic Crystal and Metasurface Architectures for Advanced Lightsails
Source: Nano Lett. 2024 May 23;24(22):6689–95. doi: 10.1021/acs.nanolett.4c01374 (PMC11157646; doi:10.1021/acs.nanolett.4c01374)
Supplement: Supplementary file 1 — nl4c01374_si_001.pdf [file nl4c01374_si_001.pdf]

Supplementary Materials: Broadband, High-Reflectivity Dielectric Mirrors at Wafer Scale: Combining Photonic Crystal and Metasurface Architectures for Advanced Lightsails

Jin Chang,[†] Wenye Ji,[‡] Xiong Yao,[†,¶,§] Arnold J. van Run,[∥] and Simon Gröblacher[*,†]

†*Kavli Institute of Nanoscience, Department of Quantum Nanoscience, Delft University of Technology, 2628CJ Delft, The Netherlands*

‡*Department of Imaging Physics, Delft University of Technology, Lorentzweg 1, 2628CJ, Delft, The Netherlands*

¶*Faculty of Physics, School of Science, Westlake University, Hangzhou 310030, P.R. China*

§*Department of Physics, Fudan University, Shanghai 200438, P.R. China*

∥*Kavli Institute of Nanoscience, Delft University of Technology, 2628CD, Delft, The Netherlands*

E-mail: s.groeblacher@tudelft.nl

Theoretical Analysis

To understand why the SiN PhC/Si bilayer membrane structure exhibits a broader reflection bandwidth compared to a single-layer SiN photonic crystal, we perform further simulations

based on the theory of multi-layered media equivalent wave impedance.[1,2] The results are presented in Fig. 1 below.

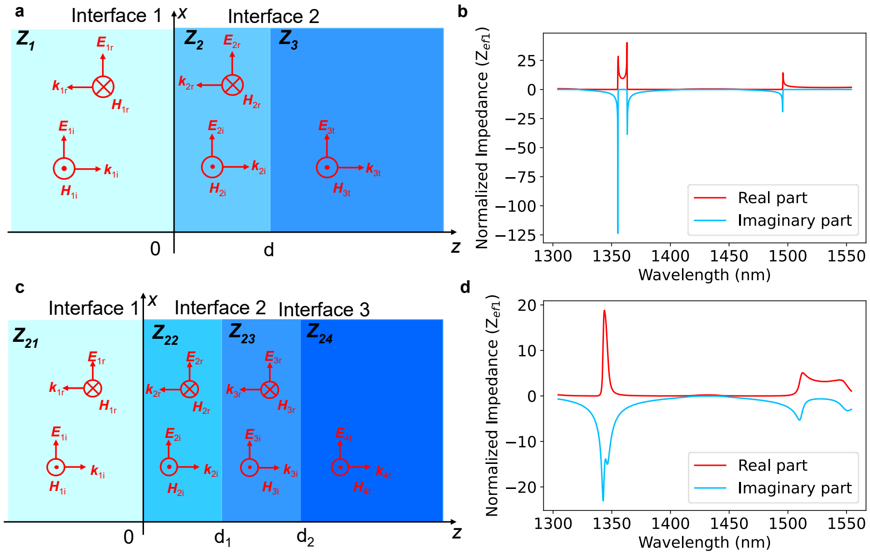

Figure 1: Multi-layered media equivalent wave impedance simulation and analysis of both SiN photonic crystal and SiN PhC/Si bilayer membrane architecture.

In Fig. 1a, going from left to right, we have three media labeled 1, 2, and 3, with their normalized impedance denoted as Z_1, Z_2, and Z_3. These three distinct lossless media have parallel interfaces at $z = 0$ and $z = d$, with the thickness of media 2 being denoted as d. When an electromagnetic wave vertically impinges from media 1 along the positive z-axis, reflections, and transmissions occur at the interfaces at $z = 0$ and $z = d$. Consequently, there are incident waves propagating in the $+z$ direction and reflected waves propagating in the $-z$ direction in both media 1 and media 2, while only transmitted waves propagate in media 3. The reflection coefficient at the interface $z = 0$ is given by

$$R_1 = \frac{Z_{ef23} - Z_1}{Z_{ef23} + Z_1}, \tag{1}$$

where Z_{ef23} represents the equivalent wave impedance of media 2 and media 3 at $z = 0$, and

$$Z_{ef23} = Z_2 \frac{Z_3 + jZ_2 \tan(\beta_2 d)}{Z_2 + jZ_3 \tan(\beta_2 d)}, \tag{2}$$

where j is the imaginary part, and $\beta_2 = \omega\sqrt{\epsilon\mu}$ is the propagation constant of the electromagnetic wave in media 2. In the case of the single-layer SiN photonic crystal structure, the SiN photonic crystal serves as media 2, with air layers both in the forward (media 1) and backward (media 3) directions, resulting in $Z_1 = Z_3 = Z_0$. Based on the reflectance coefficient of the SiN photonic crystal and Eq. (1), we can compute the normalized equivalent impedance Z_{ef23}, as depicted in Fig. 1b.

Subsequently, we consider the SiN PhC/Si bilayer membrane model. As this structure consists of two media layers, we equivalently model it as a four-layered media structure, as shown in Fig. 1c. Silicon represents media 2, and the SiN photonic crystal corresponds to media 3, with their normalized impedance denoted as $Z_{22} = Z_0\sqrt{\frac{\mu_r}{\epsilon_r}}$ and Z_{23}, respectively. The forward (media 1) and backward (media 4) directions in this case both consist of air layers, resulting in $Z_{21} = Z_{24} = Z_0$. At the interface $z = d_1$ between media 3 and media 4, the equivalent wave impedance $Z_{ef2} = Z_{ef23}$. Subsequently, according to Eq. (2), we calculate the equivalent wave impedance at $z = 0$ for media 2, 3, and 4 as

$$Z_{ef1} = Z_{22}\frac{Z_{ef2} + jZ_{22}\tan(\beta_2 d_1)}{Z_{22} + jZ_{ef2}\tan(\beta_2 d_1)}. \tag{3}$$

Finally, by applying Eq. (1), we calculate the reflection coefficients as

$$R_2 = \frac{Z_{ef1} - Z_0}{Z_{ef1} + Z_0}. \tag{4}$$

The theoretical calculation results of the reflection coefficient for the designed reflector are presented in Fig. 1d. It can be seen that the real and imaginary parts of the MPhC structure's optical impedance are significantly modified around 1500 nm, thus its high reflectance is extended into longer wavelength compared to a single-layer SiN device. Therefore, by introducing an additional Si layer and appropriately designing its thickness and photonic crystal parameters, we can achieve high-efficiency and wideband reflection.

References

(1) Stanciulescu, C.; Bobulescu, R.; Surmeian, A.; Popescu, D.; Popescu, I.; Collins, C. Optical impedance spectroscopy. *Applied Physics Letters* **1980**, *37*, 888–890.

(2) Pedrotti, F. L.; Pedrotti, L. M.; Pedrotti, L. S. *Introduction to Optics*; Cambridge University Press, 2017.
